# The engaged health sciences library liaison

**DOI:** 10.29173/jchla29539

**Published:** 2021-04-02

**Authors:** Nicole Dunnewold

**Affiliations:** Research and Learning Librarian, Health Sciences Library, University of Calgary, Calgary, AB

Alcock L, Thormodson K, editors. **The engaged health sciences library liaison**. Lanham: Rowman & Littlefield; 2020. Softcover: 194 p. ISBN: 978-1-5381-2675-2. Price: USD $55.00. Available from: https://rowman.com/ISBN/9781538126769/The-Engaged-Health-Sciences-Library-Liaison

Over the years, the role of the health sciences library liaison has expanded and evolved. *The Engaged Health Sciences Library Liaison*, through a collection of liaison program and activity descriptions, features the new opportunities associated with these changes. While the book’s editors, Lindsay Alcock and Kelly Thormodson, do not claim to be exhaustive in the innovative programs they highlight, they do seek to inspire ideas and spark discussion. True to its title, the book is aimed at health sciences library liaisons of all kinds; it also holds relevance for liaison librarians beyond the health sciences, whether they are attempting to broaden their reach or increase their impact. The book itself is not a handbook for liaison librarianship, but rather provides examples, ideas, and inspiration for liaison program design and services.

Divided into eleven chapters, the book is roughly organized based on target audiences and liaison functions. Chapter one presents a historical view of the University of Florida’s Health Science Center Libraries (UF HSCL) liaison program from 1999 to present day. While originating as a traditional unit-based liaison program, it has evolved to include functional librarians, such as a bioinformationist, whose skill sets benefit numerous units. Chapter two zeroes in on the Student Educational Enrichment Program offered at Augusta University, describing the efforts of Augusta University librarians to develop an information literacy curriculum to support it. Chapters three through five shift focus to the undergraduate medical education (UME) audience, featuring four universities’ approaches to interfacing with medical students: UF HSCL, University of British Columbia, Rowan University, and the University of North Dakota. Of particular interest is UF HSCL’s assignment of a unique liaison librarian to each cohort of medical students to support them throughout their four years of medical school. Chapter six outlines the collaboration between Memorial University health sciences librarians and the instructor of a graduate level systematic review course to provide one-on-one support to students, thereby enabling them to conduct a systematic review in a four-month time frame. Chapter seven focuses on the successful faculty collaborations developed by librarians at the Harrell Health Sciences Library (HHSL), detailing the functional and unit-specific roles of each of the nine librarians in Figure 7.1 on page one hundred and three. In Chapter eight, Heather Collins describes the unique position of health sciences library liaisons to champion interprofessional engagement, while Chapter nine reviews the rather recent phenomenon of liaison involvement in curriculum mapping, providing recommendations for initiating such involvement. Chapter ten caters to a niche audience by outlining how special collections librarians can promote and share their collections in both traditional and contemporary ways. The book is nicely rounded off with a final chapter that returns to the UME audience through clerkship engagement and addresses the Postgraduate Medical Education audience (PGME) through a robust liaison program for residents.

*The Engaged Health Sciences Library Liaison* is cleanly edited and includes content relevant to all health sciences library liaisons. Many chapters include an evaluative element, enabling individuals and teams wishing to implement specific liaison activities or programs in their own environment to learn from the lessons of the authors. While I found some chapters more engaging and applicable than others, at the close of the book, I was left feeling encouraged and inspired to expand my own role as a liaison librarian. Overall, the book provides a wealth of creative ideas for engaging with diverse user groups in the health sciences and demonstrates the ever-enduring importance of the liaison librarian role.

Though a variety of Canadian and American libraries and liaison programs are represented in the pages of *The Engaged Health Sciences Library Liaison*, certain libraries are featured multiple times. With the abundance of innovative liaison activities occurring across North American health sciences libraries, it is regrettable that more institutions are not showcased. Some readers may find this book at once motivating and discouraging, depending on the budget and resources available at their institution; smaller libraries with fewer liaisons on staff may find it difficult to implement liaison programs that meet the same level of robustness as those described in this book. As a liaison librarian serving both UME and PGME audiences, I was impressed by the number of chapters addressing UME liaison activities, but disappointed by the lack of PGME-related content. That being said, the book addresses several aspects of health sciences librarianship that are often less discussed, such as special collections promotion, curriculum mapping, and interprofessional engagement, potentially filling a gap left by other books on health sciences librarianship.

While the opportunities and changes presented to today’s health sciences library liaisons can be overwhelming and even dizzying, it is comforting to note that the key elements of an engaged liaison can be condensed into the following four categories identified by Alcock and Thormodson in their preface: “collaboration, communication, collegiality, and value”. At a foundational level, these four categories can be addressed by libraries of all sizes and librarians with any amount of experience. *The Engaged Health Sciences Library Liaison* serves as a stimulus to take the liaison librarian role to the next level. I encourage anyone interested in expanding or redesigning their liaison services to add this book to their reading list.

